# The piloting of a specific support programme for Grade R teachers on attention deficit hyperactivity disorder: The process of development

**DOI:** 10.4102/sajcd.v66i1.600

**Published:** 2019-03-26

**Authors:** Marguerite de Jongh, Anna-Marie Wium, Wilna Basson

**Affiliations:** 1 Discipline of Speech-Language Pathology and Audiology, Sefako Makgatho Health Sciences University, Ga-Rankuwa, Pretoria, South Africa; 2 Department of Psychology, Sefako Makgatho Health Sciences University, Ga-Rankuwa, Pretoria, South Africa

## Abstract

**Background:**

Attention deficit hyperactivity disorder (ADHD) is one of the most common psychiatric disorders in children and is generally identified and diagnosed during the preschool years. Preschool learners with ADHD are at risk of developing emergent literacy difficulties, crucial for the development of reading and writing. Many teachers have insufficient training to identify and address barriers to learning, such as ADHD.

**Aim:**

The aim of this article was to report on the process followed in the development of a specific support programme for Grade R teachers on ADHD and on the piloting of the programme.

**Method:**

An adapted version of the intervention research model provided the structure and phases for the development, implementation and evaluation of the support programme. Current literature on ADHD training programmes, adult learning principles and Bronfenbrenners’ ecosystemic framework was explored to develop the programme, training material, manual and method of presentation (Phase 1). Workshops were presented to 65 Grade R teachers working in an urban and semi-rural context (Phase 2). Participants were made aware of the symptoms of ADHD, and early identification and management of specific barriers to learning, such as ADHD, in order to reduce the risk of educational complications. Participants provided feedback (Phase 3) on the training, training material and manner of presentation following the workshops.

**Results:**

The results obtained in phases 1 and 2 of the adapted intervention research model included the compilation of the information presented in the workshops and the training manual, as well as the instructional phase and piloting of the programme. The results obtained for Phase 3 include a summary of the feedback provided by the Grade R teachers on how they experienced the training. Participants’ feedback confirmed that the programme was valued and that their training expectations were met.

**Conclusion:**

The intervention research model provided a valuable structure for the development and piloting of a specific support programme. This study can be replicated, and may pave the way for future support programmes for teachers. Capacity building of teachers is of the utmost importance in raising education standards in South Africa.

**Keywords:**

Attention deficit hyperactivity disorder; ADHD; emergent literacy; Grade R teachers; support programme; intervention research model; Bronfenbrenner.

## Introduction

Attention deficit hyperactivity disorder (ADHD) is defined by the American Psychiatric Association (APA) in the fifth edition of *Diagnostic and Statistical Manual of Mental Disorders* (DSM-5) as: ‘a persistent pattern of inattention and/or hyperactivity [*which*] interferes with development, has symptoms presenting in two or more settings (e.g. at home, school, or work), and negatively impacts directly on social, academic or occupational functioning’ (APA, 2013).

According to Amod, Vorster and Lazarus (2013), these patterns of inattention and/or hyperactivity consequently impact on an individual’s well-being.

This disorder is one of the most common psychiatric disorders in children, with symptoms being recognised mainly during the preschool years (Suvarna & Kamath, 2009). Preschool children with ADHD are at risk of developing emergent literacy problems (Sims & Lonigan, 2013; Walcott, Scheemaker & Bielski, 2010), which can result in academic under-achievement (Sims & Lonigan, 2013; Wessels, 2011). Attention deficit hyperactivity disorder should therefore be managed early in the preschool years in order to support these learners to attain future academic success. The effective management of ADHD is imperative in the preschool years as it affects the development of emergent literacy, specifically phonological awareness, which is a prerequisite for the development of reading and writing (Wessels, 2011).

Many preschool teachers have insufficient training to identify and address barriers to learning (Wium, 2010). Disorders such as ADHD may be a challenge to many teachers, and they often do not recognise the link that exists between ADHD and emergent literacy problems (Sims & Lonigan, 2013; Spangler & Slate, 2012). Therefore, a specific support programme was developed and piloted for Grade R teachers to recognise the signs and symptoms of ADHD and to manage the associated emergent literacy problems. This article consequently aims to contribute to the field of programme development as it describes the process of how this particular programme was developed.

## Literature review

Skills shortages and gaps in the knowledge of ADHD (Lawrence, 2012; Lopes, 2009; Perold, Louw, & Kleynhans, 2010) and associated co-morbidities (Amod et al., 2013; Murray, 2009) among Foundation Phase teachers may compromise the effective educational management of ADHD (Lawrence, 2012; Perold et al., 2010). Internationally, researchers such as Procaccini (2013) and Spangler and Slate (2012) have identified the need for training protocols to improve teachers’ knowledge on the management of ADHD.

South African inclusive education policies, such as the White Paper 6 on Special Needs (Department of Education [DoE], 2001) and the Revised National Curriculum Statement (RNCS) Grades R-9 (DoE, 2001), require that a diverse population of learners be accommodated in the classroom context. Teachers are therefore expected to meet the needs of all learners in this environment (Perold et al., 2010). In line with national policies, all learners should be able to learn and therefore be supported and provided with suitable resources to obtain academic success (Mahlo, 2011; Wium & Louw, 2015).

Primary schools in South Africa are phasing in the Grade R (reception year) as part of the Foundation Phase. Considering that the majority of Grade R teachers were trained when tertiary training was still racially and ethnically divided, the training may have been inadequate, the necessary qualifications never obtained and additional training not presented (Wium & Louw, 2013). Thus, Grade R teachers may not be well prepared for the present-day demands in the South African educational domain (Wium & Louw, 2011). Therefore, teaching Grade R learners who experience barriers to learning (Wium & Louw, 2013), such as ADHD and concomitant emergent literacy difficulties – which mostly become noticeable in the preschool or reception year (Wessels, 2011) – may be challenging for Grade R teachers, especially if they have no background in managing such challenges.

The DoE recognised a need for training to amend previous inequities, which include retraining and supporting teachers as indicated by the Minister of Basic Education as far back as 2011 (Gumede, 2017). Support for teachers will minimise the gaps in teachers’ knowledge as improved knowledge affects the management of learners with barriers to learning and consequently learner performance (Wium & Louw, 2013). Subsequently, the DoE and the Department of Basic Education implemented national education policies such as, among others, the White Paper 6 in 2001, the new National Curriculum Statement (NCS) and the RNCS in 2001, as well as the Curriculum Assessment Policy Statements (CAPS) in 2010. These were employed to assist teachers in addressing barriers to learning. However, many teachers, especially those in previously disadvantaged areas, find it difficult to implement these policies because they may not be equipped to do so (Wium & Louw, 2015). The retraining of teachers and teacher support is therefore fundamental to the successful implementation of these policy documents (Wium & Louw, 2015). Recent policy changes aim to bring about change from supporting the child with barriers, to learning to support the teacher (Wium & Louw, 2013).

Research conducted by Sims and Lonigan (2013), as well as by Spangler and Slate (2012), emphasises the fact that training programmes need to address gaps in teachers’ overall knowledge of ADHD and emergent literacy, including awareness, skills, motivation, values, accountability and attitudes (Wium, 2010). The training of teachers will consequently affect learners, helping them to succeed both socially and academically (Murray, 2009; Perold et al., 2010; Procaccini, 2013). Internationally, training programmes with a focus solely on ADHD have been conducted by researchers such as Jones and Chronis-Tuscano (2008), Syed and Hussein (2010) and Procaccini (2013). Although previous studies in the South African context described teachers’ knowledge of ADHD (Perold et al., 2010), none reported on teachers’ ability to identify and manage barriers to learning, such as ADHD and co-morbid conditions. Two local programmes aimed at Foundation Phase teachers only addressed either ADHD (Wessels, 2011) or emergent literacy (Wium, 2010). Several local researchers pointed out that quality support programmes and guidelines should be developed for teachers in the South African context (Amod et al., 2013; Olivier, Anthonissen & Southwood, 2010; Perold et al., 2010; Wium, 2010), as there is a paucity of research on the development of support programmes for preschool teachers in the identification and management of ADHD and co-morbid conditions such as emergent literacy. Findings by Girolametto, Weitzman, Lefebvre and Greenberg (2007), as well as Lessing and De Wit (2008), indicate that teachers in the local context do not adequately assist learners when co-morbidities of ADHD arise.

A need was identified for the development of a support programme for Grade R teachers in the identification and management of learners with barriers to learning, including ADHD, to prevent delays in the acquisition of pre-literacy skills. The main aim of the article is to share with the readers how a proposed support programme for Grade R teachers to facilitate knowledge on ADHD was developed and how it was perceived.

This article specifically reports on the process followed to develop such a support programme for Grade R teachers, as well as to report on the piloting of the training. The programme consisted of workshops and a corresponding training manual. The feedback provided by the participants on the training material and manner of presentation following the workshops (Phase 3) is briefly presented as the scope of the article is mainly on the development process. A detailed discussion on the feedback provided by the participants and the analysis will be outlined and presented in a follow-up article. This article therefore mainly focuses on Phase 1 of the process (Table 1), consisting of the development process; design; development of the training content and manual; manner of presentation; and the piloting of the programme (Phase 2) as presented in the methodology.

**TABLE 1:** Research process: Phases of the research.

Step	Preparation phase: Planning and approval	Phase 1: Programme development	Phase 2: Programme implementation and data collection	Phase 3: Programme evaluation: Data analysis and dissemination of results
Step 1	Permission from DBE, School Research Ethics Committee (SREC) and Medunsa Research Ethics Committee (MREC)	Review relevant literature on: ADHD Emergent literacy Adult learning Ecosystemic model	Brief district facilitator (DBE) Invite teachers to participate in the support programme	Quantitative data analysis (close-ended questions)
Step 2	Develop baseline questionnaire – Pre-training questionnaire	Develop the support programme and training manual according to the literature review indicated in Step 1	Informed consent	Qualitative data analysis (open-ended questions)
Step 3	Brief district facilitator (DBE)	Finalise questionnaires	Baseline or pre-training data collection	Qualitative data analysis (focus groups)
	Invite teachers to participate in the support programme	Finalise focus group questions	-	-
Step 4	-	-	Programme implementation	Dissemination of results Report to the DBE
Step 5	-	-	Post-training data collection	-
Step 6	-	-	Focus group discussions	-

ADHD, attention deficit hyperactivity disorder; DBE, Department of Basic Education.

## Methodology

### Programme development process (Phase 1)

The structure for the research, including the design, implementation and piloting of the support programme, was provided by the various phases described by the intervention research model. In 1994, Thomas and Rothman included six phases in their intervention research model. These are the problem analysis and project planning, information gathering and synthesis, design, early development and pilot testing, evaluation and advanced development, and dissemination of information (De Vos, Delport, Fouché & Strydom, 2011). The phases of this model were adapted and consequently renamed for the purposes of this research and provided the structure for the study.

A summary of the structure for the research is presented in Table 1.

#### Theoretical framework for the training: Ecosystemic model

The support programme was based on Bronfenbrenner’s (1979, 2005) ecosystemic model and was developed by a speech-language therapist, with a special interest and experience in ADHD, in collaboration with a psychologist, focusing on different role players in the ecosystemic framework. According to the bio-ecological model, speech-language therapists and psychologists form part of the exosystem, which also includes district facilitators, remedial and learning support teachers, and therapists and educational psychologists, among others (Mahlo, 2011). These role players, including speech-language therapists, collaborate with teachers and implement teacher support programmes. They also assist teachers with the assessment and management of learners with barriers to learning (Mahlo, 2011), as their role in the educational context includes programme design and prevention (Wium & Louw, 2013).

Bronfenbrenner (2005) states that children, in this case learners, should be viewed holistically within different social contexts. These contexts highlight the role of mutually dependent relationships between individuals and the systems that they are exposed to, as well as the structures in the environment that shape the development of a human being (Mahlo, 2011). Learners therefore do not exist in isolation, but are part of different systems. These systems should effectively work together to the advantage of all learners in schools, including those who experience barriers to learning.

This study placed emphasis on the relationship between Grade R teachers and learners with barriers to learning and how Grade R teachers could support learners with behavioural difficulties and co-morbid conditions within an inclusive environment (Denis, 2012; Lewthwaite, 2011). In addition, the interdependency between ADHD and associated educational problems within a broader social ecological model was also highlighted (Lopes, 2009). The training stressed the relationship and responsibilities of different role players within the educational context. These role players in the educational management of ADHD include teachers, parents and peers, school governing bodies, the Department of Basic Education, school districts and so on. These role players are considered representatives of Bronfenbrenner’s (1979, 2005) ‘meso-’ and ‘exosystems’ working together in implementing policies and ensuring that all learners with ADHD receive adequate support (Lewthwaite, 2011).

#### Process of content development

**FIGURE 1:**
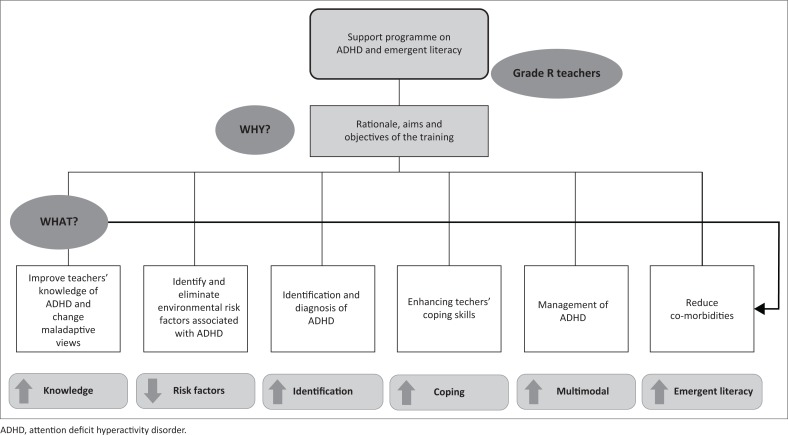
Components of the support programme on attention deficit hyperactivity disorder and emergent literacy.

A literature review (including a Google Scholar search between 2008 and 2015) that focused on previous training programmes for teachers (both internationally and in the South African context) on ADHD and the consequent development of early literacy, as well as recommendations offered by these researchers in the field of ADHD, provided guidelines for the development of the support programme (Amod et al., 2013; Jones & Chronis-Tuscano, 2008; Perold et al., 2010; Procaccini, 2013; Syed & Hussein, 2010; Wessels, 2011; Wium, 2010). These recommendations obtained from the literature search indicated that the following need to be addressed throughout the training; that is, general knowledge on ADHD; the early identification of behaviour associated with ADHD and the management of ADHD. The basic outline of the support programme, as a result of the literature review, is presented in Figure 1. This specific support programme is, however, unique as it focuses on the early identification and management of barriers to learning, including both ADHD and emergent literacy, and is likely the first of its kind to be implemented for Grade R teachers in the South African context.

#### Training content

As a *result* of the process followed, two main themes were identified for the training, which included ADHD and emergent literacy. The ADHD theme included different topics such as defining ADHD; the features, prevalence and aetiology; the symptoms and risk factors associated with ADHD; the co-morbid conditions related to ADHD; the risk factors aggravating the condition; the identification and diagnosis of ADHD; and coping strategies for teachers managing ADHD, as well as the multimodal treatment of ADHD including pharmacological and non-pharmacological interventions. The emergent literacy topics comprised the impact of ADHD on emergent literacy and reducing the risk of educational complications such as emergent literacy. An improved knowledge on the above will affect teachers’ management and support of learners with ADHD and co-morbid barriers to learning.

Additionally, the content of the support programme is sequenced in such a way that the simple or less complicated information is presented before the more complex information, as implied in Figure 1. The components such as the facilitation of general knowledge on ADHD is presented first, after which the identification and management of this barrier to learning is offered. Finally, the figure emphasises the interdependency between ADHD and co-morbidities, which infers that the risk of further educational complications might be reduced should teachers’ knowledge on early identification and referral improve.

#### Training manual

A training manual was developed as a resource guide and corresponds with the training content and the information provided in the workshops. The manual comprised the rationale for the training, newspaper clippings, case scenarios and PowerPoint slides as well as material to be used in the classroom context. Information on the identification and management of specific barriers to learning, including ADHD and emergent literacy problems, as well as the training process and training content presented in Figure 1 and Table 3, was similarly included in this resource manual. A directory of services available, such as the relevant role players for the identification, diagnosis and management of ADHD, was also incorporated into the manual. The manual was handed to the Grade R teachers during the training.

#### Design of the training component and manner of delivery

The workshops comprised training, as well as interactive components that had opportunities for further practice in the classroom context in order to support the Grade R teachers. These included the initial identification and, as a result, the management of specific barriers to learning, such as ADHD and emergent literacy problems. The identification of barriers to learning is recognised as one of the seven roles of educators (Rossouw, 2009). Consequently, the use of handouts, newspaper clippings, PowerPoint slides and icebreakers; the presentation of case studies and real-life scenarios; small group discussions; group work; problem-solving activities; and roleplay, among others, were included in the training.

Considering that teachers are adult learners, the training was additionally designed in accordance with adult learning principles, as suggested by the adult learning theories such as Andragogy, originally proposed by Knowles (1975, 1984). Consequently, additional theories of adult learning (such as experiential learning, self-directed learning, transformative learning as well as the principles underlying these theories), the factors that may influence adult learning, and the way all these aspects influence learning were explored and integrated to provide the underlying basis of the support provided (Conlan, Grabowski & Smith, 2012; Freire, 2004; Killen, 2012; Kolb & Kolb, 2005; Wessels, 2011). The underlying principles also recognise that adults are internally motivated and that their self-concept changes from being dependent learners to being self-directed learners. Furthermore, as adult learners accumulate experience, these experiences become resources for obtaining added knowledge, which can then be incorporated and applied to work-related tasks and problem-solving (Knowles, 1984).

Additionally, these principles also suggest that training is most effective when groups are smaller; training is applied for shorter periods and, where possible, is preventative in nature. Recommendations provided by researchers such as Wium (2010) and Wessels (2011) also suggest that training should be interactive and collaborative, that the needs of the teachers should be addressed and that the support and knowledge of the teachers should be integrated and transferred into the school context.

The stated theories, principles and recommendations provided guidelines for the development of and the manner in which the workshops were delivered. The training was therefore designed around and based on various adult learning theories and in accordance with the adult learning principles, and not ‘the way … [*they*] … were [*initially*] taught’ (Zoller & Harrison, 2007, p. 1). As a result, the training was interactive and the Grade R teachers were dynamic participants and in control of their own learning (Killen, 2012; Wessels, 2011). These principles, as integrated into the teaching and learning strategies, are reflected in Table 2. The learning strategies included hands-on tasks performed by the Grade R teachers, such as completing checklists related to simulated cases. An outline of the support programme, the sub-aims, specific outcomes, assessment criteria and activities, including the teaching and learning strategies, as well as the different topics offered, is presented in Table 2.

Additionally, Table 2 provides a summary of the workshop content and procedures presented to the teachers to facilitate their understanding of ADHD and emergent literacy. An improved understanding of ADHD will support the Grade R teachers’ identification of ADHD as they utilise identification procedures such as rating scales and checklists in the classroom context. In addition, the accurate identification of ADHD will enable the Grade R teachers to manage the disorder fittingly. The management may include parental discussions and referrals to relevant allied health and medical professionals for further investigation of Grade R learners’ behaviour and attention.

The outline of the programme, the different sessions and the themes and topics presented is shown in Table 3. Table 3 aligns with the information presented in Table 2 as well as with the information provided in the training manual.

After the development of the programme content and the manual, the programme was piloted (Phase 2), as shown in Table 1.

### Piloting of the programme (Phase 2)

The programme was presented as two workshops to 65 Grade R teachers. These teachers represented 57 schools in a specific district of Tshwane. Two groups of participants were identified; that is, one group with Grade R teachers from urban schools and another group with Grade R teachers from a semi-rural context. Two district facilitators, representing the Department of Basic Education from the specific district, also attended the training and assisted in the facilitation of the training.

The training consisted of two half-day workshops for the urban school participants and one full-day workshop for the participants from a semi-rural context. The outline of the programme and topics presented are indicated in Tables 2 and 3, as discussed in the section ‘Design of the Training Component’. The aim of the training was to make participants aware of the symptoms of ADHD and the importance of early identification and management of specific barriers to learning such as ADHD in order to reduce the risk of educational complications. Following the implementation of the training (Phase 2), the participants provided feedback on the training (Phase 3), as shown in Table 1.

**TABLE 2:** Outline of the support programme: Aims, outcomes, assessment criteria, activities, teaching and learning strategies, and assessment strategies.

Sub-aims (What the researcher intends to accomplish with the training)	Specific outcomes (SOs) (What the Grade R teachers are expected to know, understand and implement after the training)			Assessment criteria (AC) (Manner in which to determine if the SO was met)			Activities (How the aims and activities will be met)	Teaching and learning strategies (How?)
Aim 1	SO1:	SO2:	SO3:	AC1:	AC2:	AC3:		
To improve the participants’ general knowledge and understanding of the following topics:	Identify the factors that contribute to and are associated with ADHD	Identify the different steps in the assessment and identification process	Select and recommend appropriate learning material for emergent literacy (phonological awareness)	Grade R teachers demonstrate improved understanding by reflecting on their:	Grade R teachers demonstrate improved understanding by reflecting on:	Grade R teachers display improved understanding by reflecting on:	A 20-min group discussion before presentation	Thematic learning
ADHD and emergent literacy	Identify the characteristics or symptoms of ADHD in Grade R learners	Select appropriate identification procedures for Grade R learners with ADHD	Demonstrate the relationship between the strategies presented in the White Paper 6 on the management of ADHD in the classroom and literacy outcomes (DoE, 2001) and link with the outcomes of the support programme	Knowledge of ADHD (factors)	The assessment and identification process for ADHD	The relationship between ADHD and emergent literacy	PowerPoint presentation, video clip and group discussion on aetiology and symptoms of ADHD and link with case study provided	Direct instruction
Ability to select and apply appropriate identification or assessment procedures	Identify the relationship between ADHD and co-morbidities (emergent literacy)	-	Identify, select and recommend team members in the management of learners with ADHD and associated emergent literacy problems	Characteristics and symptoms in learners associated with ADHD	Demonstrate how the DSM-5, checklists and rating scales can be utilised in the identification of learners with ADHD	The management of ADHD and emergent literacy (post-training)	PowerPoint presentation and group discussion on the relationship between ADHD and emergent literacy	Direct instruction
Management of ADHD and emergent literacy (phonological awareness)	-	-	Demonstrate how the learning environment can be adapted	The relationship between ADHD and emergent literacy development	-	The role of different team members in the management of ADHD and emergent literacy problems	Presentation on identification procedures	Hands-on tasks
Carry over into the classroom context and other ‘systems’; that is, home environment	-	-	-	-	-	Learning environment adaptations in the management of learners with ADHD and associated emergent literacy problems	Application and group discussion on the use of rating scales, checklists and DSM-5	Feedback
-	-	-	-	-	-	-	Presentation of identified programme for the management of emergent literacy: 20 min	Applied learning
-	-	-	-	-	-	-	Presentation and group discussion on inter-relationship between DoE and support programme outcomes	Direct presentation
-	-	-	-	-	-	-	Presentation and group discussion on the role of different team members	Hands-on tasks
-	-	-	-	-	-	-	Presentation and group discussion on learning environment adaptations: link with case study	Feedback
-	-	-	-	-	-	-	-	Self-directed learning

ADHD, attention deficit hyperactivity disorder; DSM-5, Diagnostic and Statistical Manual of Mental Disorders.

**TABLE 3:** Outline of the sessions, themes and topics of the workshop.

Sessions	Variable	Outline
Session 1 (Part 1)	Theme	ADHD
	Topics	A: Introduction: Defining ADHD
		B: ADHD: Features, prevalence, aetiology and symptoms (including co-morbidities)
		C: Background on environmental risk factors associated with ADHD: Dietary factors Nutritional deficiencies Biological factors Abnormal lighting Exposure to environmental toxins Psychosocial factors
		D: Identification and diagnosis of ADHD: Predominantly hyperactive-impulsive presentation Predominantly inattentive presentation Combined presentation
Session 2 (Part 2)	Theme	ADHD
	Topics	E: Coping with ADHD (strategies for teachers)
		F: Multimodal treatment for management of learners with ADHD: Pharmacological interventions Non-pharmacological interventions: ■ Dietary modifications ■ Psychosocial education ■ Behavioural modification ■ Adapt the school environment ■ Classroom strategies (White Paper 6)
Session 3 (Part 3)	Theme	Emergent literacy
	Topics	G: Impact of ADHD on co-morbid conditions, for example, emergent literacy or related emergency literacy problems
		H: Reducing the risk of further complications or co-morbid conditions
		I: Support for learners with emergent literacy problems

ADHD, attention deficit hyperactivity disorder.

### Participants’ feedback (Phase 3)

Qualitative data were obtained from questionnaires and two focus group discussions. These provided information on how the participants perceived the training and the content that was provided, the training material and the manner of presentation following the workshops. Participants had to complete the open-ended questions as presented in the last section of the post-training questionnaire. Two focus group discussions were held, one for the urban and one for the semi-rural context.

The responses to the open-ended questions and information obtained in the focus group discussions were grouped; themes were identified and then analysed. The researcher made use of inductive analysis (Creswell, 2009) by organising the data into increasingly more abstract units of information in order to ascertain how the participants experienced the training.

## Ethical considerations

Ethical clearance to perform the research was obtained from the Sefako Makgatho Health Sciences University Research and Ethics Committee (MREC/M/336/2014:PG). Permission was obtained from the Department of Basic Education to conduct research in a specific district and the Grade R participants provided consent to participate in the study.

## Results and discussion

The results are presented in accordance with the different phases (phases 1, 2 and 3) of the intervention research model.

### Phases 1 and 2

The structure, development and implementation of the programme formed part of the results for phases 1 and 2. The outcomes of phases 1 and 2, consisting of the instructional design, process of development, training content, training manual, manner of delivery and implementation of the programme at the workshops, are presented as part of the methodology section and are considered the results for Phase 1 (programme development) and Phase 2 (programme implementation and data collection). These results for phases 1 and 2 are also presented in Tables 1, 2 and 3.

### Phase 3

The results obtained in Phase 3 are related to the participants’ perceptions of the programme content and feedback on how they experienced the training. The feedback included Grade R teachers’ opinions on the workshops, training material and manner of presentation, as well as suggestions on how to advance the programme for future implementation. In this article, only a summary of the open-ended feedback in the post-training questionnaire and the feedback regarding the teachers’ experiences and opinions, as obtained from two focus group discussions, are presented as results for Phase 3.

The qualitative feedback from the participants in the questionnaire indicated that they were grateful for and positive about the training. Similar themes were identified from the focus group discussions. The two main themes included ‘Value of the programme’ and ‘Participants’ suggestions and recommendations’. The focus group participants likewise indicated that they were appreciative of and were more confident in identifying and managing ADHD after the training. They also stated that the training was both beneficial and effective, delivered results efficiently and generally met their expectations. The feedback included comments such as:

‘It was fantastic … [*it*] held my attention … the presentation was fun so I felt I could participate … I wasn’t bored at all [*because*] you used a lot of … examples.’ (Female, Grade R teacher, urban context)

‘You took our needs into consideration.’ (Female, Grade R teacher, urban context)

‘We really have those kinds of children but no idea how to deal with them … it opened up a new world.’ (Female, Grade R teacher, semi-rural context)

These comments echoed the importance of integrating adult learning principles in the execution of such programmes.

The feedback from the participants also emphasised that their awareness improved regarding the role of mutually dependent relationships between individuals and the systems that they are exposed to (Bronfenbrenner, 1979, 2005). They stressed the interaction between different role players, such as parents and teachers who should be equally responsible in improving the academic outcomes of learners with ADHD. Comments including the following:

‘I really had a positive experience … I can contribute more … and positively … influence the parents’. (Female, Grade R teacher, urban context)

and providing evidence in this regard:

‘… if you engage the parent, you will see the parent also giving the child support at home.’ (Female, Grade R teacher, semi-rural context)

The participants furthermore revealed that an additional advantage of the support was that the training material; that is, manual, was valuable and should also be used in follow-up training. One of the participants stated:

‘I have something to show … and it was nice for me to make copies from the manual’. (Female, Grade R teacher, urban context)

Another participant added:

‘I can add the checklist … as part of the report’. (Female, Grade R teacher, urban context)

They reflected that the manual could be utilised as a resource guide for teachers in the identification and management of specific barriers to learning, such as ADHD.

The feedback also yielded suggestions for the enhancement of knowledge concerning ADHD. These included the need for additional training, not only for Grade R teachers but for all Foundation Phase teachers and other stakeholders involved. Furthermore, they requested that the Department of Basic Education be involved in the follow-up training on ADHD. Topkin, Roman and Mwaba (2015) suggest that training should not be once-off but continuous in order for teachers to improve their knowledge by solving actual problems that arise in the classroom context.

Additional feedback from the participants indicated that the information conveyed about the misconceptions of ADHD as well as the information on psycho-stimulant medication was particularly useful and should be included in future training. They restated the importance of an integrated approach, including the different role players in the management of ADHD and emergent literacy, and the significance of this information in training.

The feedback and recommendations from the participants also provided information on how to further develop the programme for future implementation, as suggested by Patton in 2008.

Participants reiterated that interactive components such as demonstrations, role-play and problem-solving activities should be expanded in future training. The restricted amount of interactive components as perceived by the participants can be regarded as a limitation of the programme. Furthermore, the fact that only teachers from one district participated in the study can also be considered a limitation as participants expressed the need for additional training. It is therefore recommended that this support programme be repeated in more districts and may need to be adjusted as contexts may differ significantly.

## Conclusion

The complex nature of ADHD, the intricacies of the condition in the South African context and skills shortages, as well as the lack of training for teachers provided the rationale for this research. Spaull (2013) affirmed that the support of teachers should be seen as a lifelong investment in their skills in order to assist in the development of learners who exhibit barriers to learning. The implementation of such support programmes for teachers – to build capacity and raise education standards – is of the utmost importance in the current South African context.

The study stressed the usefulness of the intervention research model as a framework for the development and imple-mentation of the support programme for Grade R teachers. The positive feedback obtained from the Grade R participants provided evidence that the training was valued and that teachers needed such information on ADHD. Thus, the intervention research model was suitable for the development of support programmes and can therefore be replicated for the purpose of developing similar support programmes. It will most possibly ‘serve the needs of education and its components within a broader systemic approach’ (Wium, 2010:4-3).

## References

[CIT0001] American Psychological Association (APA) (2013). *Diagnostic and statistical manual of mental disorders* (*DSM-5*^*®*^). Washington, DC: APA.

[CIT0002] Amod Z., Vorster A., & Lazarus K (2013). Attention-deficit/hyperactivity disorder (ADHD) as a barrier to learning & development within the South African context: The perspective of teachers In Banerjee S. (Ed.), *Attention deficit hyperactivity disorder in children and adolescents*. Rijeka, Croatia: Intech.

[CIT0003] Bronfenbrenner U (1979). *The ecology of human development*. Cambridge. MA: Harvard University Press.

[CIT0004] Bronfenbrenner U (2005). *Making human beings human: Bioecological perspectives on human development*. Thousand Oaks, CA: Sage.

[CIT0005] Conlan J., Grabowski S., & Smith K (2012). *Adult learning: Emerging perspectives on learning*. Department of Educational Psychology and Instructional Technology, University of Georgia Retrieved from http://www.attend.org.uk/sites/default/files/M1-R3A2%20Adult%20Learning%20-%20Emerging%20Perspectives%20on%20Learning.pdf

[CIT0006] Creswell J. W (2009). *Research design: Qualitative, quantitative and mixed methods approaches* (3rd edn). Thousand Oaks, CA: Sage.

[CIT0007] Denis C. M (2012). Attention deficit hyperactivity disorder and Bronfenbrenner’s Ecology of human development. *Electronic Theses & Dissertations*, 64 Retrieved from https://digitalcommons.georgiasouthern.edu/cgi/viewcontent.cgi?article=1064&context=etd

[CIT0008] Department of Education (DoE) (2001). *Education White Paper 6: Special needs education: Building an inclusive education and training system*. Pretoria: Department of Education Retrieved from http://www.polity.org.za/govdocs/whitepapers/educ6.html

[CIT0009] De Vos A. S., Delport C. S. L., Fouché C. B., & Strydom H (2011). *Research at grass roots: A primer for the social science and human professions* (4th edn). Pretoria: Van Schaik.

[CIT0010] Freire P (2004). *Pedagogy of indignation: Series in critical narratives*. Boulder, CO: Paradigm.

[CIT0011] Girolametto L., Weitzman E., Lefebvre P., & Greenberg J (2007). The effects of in-service education to promote emergent literacy in child care centres: A feasibility study. *Language, Speech, and Hearing Services in Schools*, 38(1), 72–83. 10.1044/0161-1461(2007/007) 17218537

[CIT0012] Gumede M (2017, July 31). Teacher training in spotlight. *Sowetan Live*. Retrieved from https://www.sowetanlive.co.za/news/2017-07-31-teacher-training-in-spotlight/

[CIT0013] Jones H. A., & Chronis-Tuscano A (2008). Efficacy of teacher in-service training for attention-deficit hyperactivity disorder. *Psychology in the Schools*, 45(10), 918–929. 10.1002/pits.20342

[CIT0014] Killen R (2012). *Effective teaching strategies: Lessons from research and practice* (6th edn). Melbourne: Cengage Learning Australia.

[CIT0015] Knowles M (1975). *Self-directed learning: A guide for learners and teachers*. Chicago, IL: Follet.

[CIT0016] Knowles M. S (1984). *Andragogy in action: Applying modern principles of adult education*. San Francisco, CA: Jossey Bass.

[CIT0017] Kolb A. Y., & Kolb D. A (2005). Learning styles and learning spaces: Enhancing experiential learning in higher education. *Academy of Management Learning & Education*, 4(2), 193–212. 10.5465/amle.2005.17268566

[CIT0018] Lawrence M (2012). *The issues and challenges that foundation phase educators experience when teaching learners with Attention Deficit Hyperactivity Disorder (ADHD)*. Doctoral dissertation Durban: University of KwaZulu-Natal.

[CIT0019] Lessing A., & De Witt M. W (2008). *Do teachers know what the essential literacy skills are?* Paper presented at Laying Solid Foundations for Learning meeting, Makopane, Limpopo, 30 September–1 October 2008.

[CIT0020] Lewthwaite B (2011). *University of Manitoba Centre for Research in Youth, Science Teaching and Learning: Applications and utility of Urie Bronfenbrenner’s bio-ecological theory* (pp. 3–14). University of Manitoba Retrieved from http://researchonline.jcu.edu.au/29719/

[CIT0021] Lopes M. A (2009). *South African educators’ experiences of learners who may have ADHD in their classrooms*. Doctoral dissertation Pretoria: University of Pretoria.

[CIT0022] Mahlo F. D (2011). *Experiences of learning support teachers in the foundation phase with reference to the implementation of inclusive education in Gauteng*. Doctoral dissertation Pretoria: University of South Africa.

[CIT0023] Murray E. K (2009). *Managing attention deficit hyperactivity disorder in schools: What teachers and parents believe and know*. Unpublished doctoral thesis Perth: Murdoch University.

[CIT0024] Olivier A., Anthonissen C., & Southwood F (2010). Literacy development of English language learners: The outcomes of an intervention programme in Grade R. *South African Journal of Communication Disorders*, 57(1), 58–65. 10.4102/sajcd.v57i1.50 21329267

[CIT0025] Patton M. Q (2008). *Utilization-focused evaluation*. Thousand Oaks, CA: Sage.

[CIT0026] Perold M., Louw C., & Kleynhans S (2010). Primary school teachers’ knowledge and misperceptions of attention deficit hyperactivity disorder (ADHD). *South African Journal of Education*, 30(3), 457–473. 10.15700/saje.v30n3a364

[CIT0027] Procaccini J. C (2013). *Development and assessment of an accelerated AD/HD training for teachers in elementary schools*. Doctoral dissertation: Union Institute and University, Ohio.

[CIT0028] Rossouw D (2009). Educators as action researchers: Some key considerations. *South African Journal of Education*, 29(1), 1–16. 10.1590/S0256-01002009000100001

[CIT0029] Sims D. M., & Lonigan C. J (2013). Inattention, hyperactivity, and emergent literacy: Different facets of inattention relate uniquely to preschoolers’ reading-related skills. *Journal of Clinical Child and Adolescent Psychology*, 42(2), 208–219. 10.1080/15374416.2012.738453 23186142 PMC3586984

[CIT0030] Spangler J. M., & Slate J. R (2012). Elementary school teacher perceptions of attention deficit hyperactivity disorder. *Mercer Journal of Educational Leadership*, 1(1), 1–28.

[CIT0031] Spaull N (2013). *South Africa’s education crisis: The quality of education in South Africa 1994–2011*. Johannesburg Centre for Development and Enterprise, Johannesburg, p. 1–65.

[CIT0032] Suvarna B. S., & Kamath A (2009). Prevalence of attention deficit disorder among preschool age children. *Nepal Medical College Journal*, 11(1), 1–4. 19769227

[CIT0033] Syed E. U., & Hussein S. A (2010). Increase in teachers’ knowledge about ADHD after a week-long training program: A pilot study. *Journal of Attention Disorders*, 13(4), 420–423. 10.1177/1087054708329972 19474460

[CIT0034] Topkin B., Roman N. V., & Mwaba K (2015). Attention Deficit Disorder (ADHD): Primary school teachers’ knowledge of symptoms, treatment and managing classroom behaviour. *South African Journal of Education*, 35(2), 01–08. 10.15700/saje.v35n2a988

[CIT0035] Walcott C. M., Scheemaker A., & Bielski K (2010). A longitudinal investigation of inattention and preliteracy development. *Journal of Attention Disorders*, 14(1), 79–85. 10.1177/1087054709333330 19602706

[CIT0036] Wessels E (2011). *Teacher knowledge and implementation of phonological awareness in Grade R*. Doctoral dissertation North West University: Potchefstroom.

[CIT0037] Wium A. M (2010). *The development of a support programme for foundation phase teachers to facilitate listening and language for numeracy*. Doctoral dissertation Pretoria: University of Pretoria.

[CIT0038] Wium A. M., & Louw B (2011). Teacher support: An exploration of how Foundation Phase teachers facilitate language skills. *South African Journal of Communication Disorders*, 58(2), 86.

[CIT0039] Wium A. M., & Louw B (2013). Revisiting the roles and responsibilities of speech-language therapists in South African schools. *South African Journal of Communication Disorders*, 60(1), 31–37. 10.4102/sajcd.v60i1.8 25158371

[CIT0040] Wium A. M., & Louw B (2015). CAPS: Implications for collaboration between teachers and speech-language therapists working in schools. *South African Journal of Childhood Education*, 5(1), 19–41. 10.4102/sajce.v5i1.348

[CIT0041] Zoller K., & Harrison B (2007). Sierra Training Associates: We can teach the way we were taught, or we can teach the way people learn. Retrieved from https://www.sierra-training.com/pdf/adultlearning.pdf

